# Exploring Readiness for Change: Knowledge and Attitudes towards Family Violence among Community Members and Service Providers Engaged in Primary Prevention in Regional Australia

**DOI:** 10.3390/ijerph16214215

**Published:** 2019-10-30

**Authors:** Monica Puccetti, Heath Greville, Margie Robinson, Daphne White, Lennelle Papertalk, Sandra C. Thompson

**Affiliations:** 1 Western Australian Centre for Rural Health, University of Western Australia, Geraldton, WA 6530, Australia; monicap@desertblueconnect.org.au (M.P.); Heath.greville@uwa.edu.au (H.G.); Margie.robinson@uwa.edu.au (M.R.); Lenny.papertalk@uwa.edu.au (L.P.); 2 Desert Blue Connect, Geraldton, WA 6530, Australia

**Keywords:** family violence, intimate partner violence, domestic violence, violence against women, primary prevention, community attitudes, readiness for change, community development, gender equity, community education

## Abstract

Community efforts at the primary prevention of family violence (FV) involve changing values, structures and norms that support gender inequality. This study examines the attitudes of a group of highly engaged community leaders and service providers involved in FV primary prevention in Geraldton, a small city in regional Western Australia. The outcomes of focus group discussions were mapped against a readiness for change model. Despite considerable involvement in discussions of FV prevention over time, the readiness level of these engaged community members for taking leadership roles in the prevention strategy were between pre-planning and preparation stages, although some individuals’ understanding of the drivers of FV and readiness for implementing change was higher. Key areas for further education are the role of gender inequality as the primary driver of FV, particularly rigid gender roles and men’s control of decision making, and the role of alcohol and drugs as reinforcers but not primary drivers of FV.

## 1. Introduction

Violence against women and family violence (FV) are widespread problems with lasting negative impacts on mental health and the quality of life, as well as an increased risk of serious injury and/or death [1]. Women experiencing FV are more likely to sustain physical injuries and to engage in health-compromising activities such as smoking, low physical activity, and excessive alcohol consumption than women not experiencing FV [2]. In Australia, it has been reported that one in six women have experienced physical or sexual abuse by a former or current partner, and FV is the single largest risk factor for death, disability, and injury for women aged 18–44 years, making this a high priority public health problem and a strongly gendered problem [3]. 

Australia’s National Plan to Reduce Violence against Women and their Children 2010–2022 (11) (National Plan) has a strong focus on prevention by addressing community attitudes and social norms that drive FV. Higher rates of FV occur in regional and remote areas, and rural women are more likely to have difficulty accessing services [4,5,6]. While there have been efforts to understand how the drivers of FV are perceived in a regional/rural setting, few studies have been published, with none from Western Australia (WA) [7,8]. 

This exploratory study focused on understanding the attitudes towards and knowledge of FV in a highly engaged community subpopulation in Geraldton, a regional WA city where a primary prevention strategy, the Community, Respect and Equality (CRE) Strategic Plan, had been developed [9] and was at the early stages of implementation. The CRE Plan was developed through community engagement and consultation, and it was spearheaded by two organizations (both of which were concurrently going through a process of amalgamation into one organization) which focus on women’s health, sexual health (including sexual assault), and family violence. The main focus of the CRE Plan at the time of data collection was on the CRE Agreement for Businesses and Organizations, which aimed to (i) harness momentum for the businesses and organization to address the practices, attitudes, norms and behaviors that underpin violence against women within the workplace; (ii) achieve commitment to agreed values and codes of behavior and actions in the long term; and (iii) develop corporate responsibility to take action because FV is a key social and health issue impacting employees. The first stage of the CRE Agreement was to engage local organizations and workplaces in educating their staff about FV prevention.

Geraldton is a regional center of 35,000 people in WA, with nearly one-quarter of the population born overseas and around 9.7% of the population identifying as Aboriginal. Family violence in the Greater Geraldton area is recognized as a significant problem, with the regional Police Family and Domestic Violence Response Team attending an average of 234 family violence incidents per month for a total of 2819 incidents in the 2016/2017 financial year [9]. These figures are the second highest in Western Australia, with only Kimberley, a remote region of WA, experiencing more violence per capita. Police data show the rate of assault by a family member in the city of Greater Geraldton in the period July 2018–June 2019 was 47% higher than the rate for regional WA and nearly three times the state average [10]. It is known that figures for FV under-report the true incidence, as an estimated 80% of family violence incidents go unreported to police for a variety of reasons including a fear of retaliation from the perpetrator, a lack of understanding that what is occurring is family violence, and/or a lack of trust in response services [11].

### 1.1. Definition, the Drivers of FV and Primary Prevention

This study uses the term FV, the definition used in the CRE Strategic Plan. The term FV refers not only to violence experienced within an intimate partner relationship but also within the broader context of the family, including extended family, kinship, and community networks [12,13]. It is considered more culturally inclusive and is the preferred term in Aboriginal communities [12]. 

To date, the majority of research on FV in Australia has focused on responses to violence. In recent years, tragic occurrences of FV have given this issue a high profile. The resulting confluence of government focus on the issue—from the creation of the National Plan to the Royal Commission into FV in Victoria [14,15]—has led to a growing push to understand and work towards the primary prevention of FV. The organizations Our Watch and Australia’s National Research Organization for Women’s Safety (ANROWS) arose out of the National Plan and identified gender inequality as the underpinning cause of FV. Subsequently, the Change the Story framework was created to understand and publicize the drivers (causes) of FV and pathways to primary prevention of FV [13]. Primary prevention, a relatively new area of practice and research for FV, targets the whole population [4,12,13,14,15] and requires the design and implementation of multi-level interventions. Promising primary prevention interventions target the gendered drivers of FV through a theory-based, intersectional understanding of power dynamics, through the creation of multi-sectoral and multi-level change, and through engaging the whole community in equality-based activism [16].

Consistent with international peer-reviewed research linking higher rates of worldwide FV to higher levels of gender inequality, the Change the Story framework has utilized international evidence and identified four gendered drivers that create the necessary social context for FV to occur [13,16,17,18]: (1) the condoning of violence against women, (2) men’s control of decision-making and limits to women’s independence in public and private life, (3) rigid gender roles and stereotyped constructions of masculinity and femininity, and (4) male peer relations that emphasize aggression and disrespect towards women [13]. In addition to these primary drivers, the Change the Story framework has identified factors which reinforce or exacerbate these drivers: the condoning of violence in general; the experience of and exposure to violence; the weakening of pro-social behavior, especially the harmful use of alcohol; socio-economic inequality and discrimination; and backlash factors that can occur when male status or power is challenged. These reinforcing factors can all increase the levels and severity of FV. In recognition of the history of violence and colonization perpetrated against Aboriginal and Torres Strait Islander people, as well as ongoing oppression, incarceration and disadvantages, the Change the Story framework uses an intersectional framework as a companion resource to understand additional considerations for FV in Aboriginal communities and the interaction of these factors with the gendered drivers. 

### 1.2. Community Readiness for Change 

Community readiness for change has been recognized as an important factor in the success or failure of population-level interventions, especially interventions aimed at changing deep-seated social norms and cultural attitudes. If an intervention is not targeted to the readiness level of the community, it is likely to fail. Moreover, despite good intentions, it may cause resistance and backlash to change, thereby hindering further prevention efforts [19,20]. This was taken into account in the development of the CRE Strategic Plan with the phases of the Plan being aligned to the diffusion of innovation theory in a bid to increase broader community engagement with the core messages of the Plan and reduce the potential for backlash [9].

**Figure 1:**
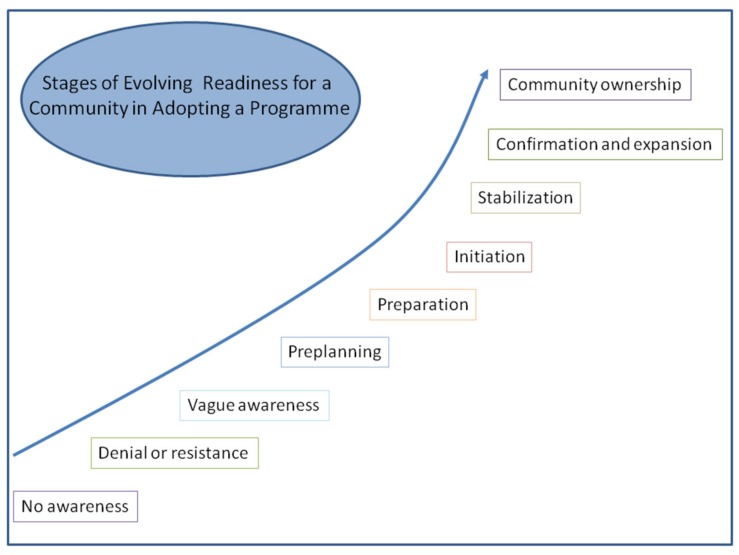
Community readiness model from the Tri-Ethnic Centre for Prevention Research in the University of Colorado [21].

The community readiness model (CRM), developed by the Tri-Ethnic Centre for Prevention Research in the University of Colorado [21], conceptualizes nine stages of community readiness for change (Figure 1). The CRM grew out of two research theories: (1) Prochaska and Velicer’s transtheoretical model, a theory of individual psychology looking at the stages an individual goes through in behavior change [22]; and (2) the social action process of community development that identifies various stages for collective action [19].

The CRM has been used to assess community readiness for FV prevention in one Australian setting—regional New South Wales (NSW) [8]. However, community assessments of readiness for change to reduce FV have not occurred in WA, a much less populated and remote state than NSW. Given that they are essential components of readiness for family violence prevention intervention, this qualitative study explores community attitudes towards and knowledge of family violence prevention in Geraldton, Western Australia.

## 2. Methods 

Focus groups with key informants were utilized to assess attitudes and knowledge about FV. Focus groups were chosen as the main data collection method to explore both individual and group attitudes, as well as how these are expressed or not expressed [23,24]. Discussions around difficult topics such as FV can be affected by social desirability bias, which is the desire to answer questions so as to be viewed favorably by others. While social desirability bias is less likely to occur in individual interviews, this study sought to understand organizational readiness for change, making it appropriate to study attitudes in a group environment [25]. For some people, discussions about FV and violence against women are more comfortably conducted in gender-specific groups [14,26]. The focus group questions (Appendix A) focused on participants’ knowledge of the CRE Plan, drivers of FV, the way in which the success of the primary prevention of FV could be measured, and what role they saw they had in implementing the CRE Action Plan in their workplace. Questions about personal experiences of FV were not included due to the sensitivity of these issues and the study’s focus on community attitudes that create or prevent family violence-condoning environments. 

Sampling was purposive in order to access the attitudes and knowledge of community members and service providers who were already highly engaged in the CRE Strategy Plan. Participants needed to be engaged in FV prevention or response, be an organizational stakeholder in the CRE Plan, or be a community leader who had been engaged with the CRE Plan development. Thus, the views were those of a highly selective subpopulation of the community, and the pool of potential participants was small.

The focus group meetings were set up in advance. All went through a process of formal and deliberative consent with the facilitator making clear the purpose of the focus group and that it was being recorded, setting clear ground rules to ensure only one person spoke at a time, encouraging discussion so that all participants had the opportunity to speak, and stressing confidentiality and that there were “no wrong answers.” 

Key organizational stakeholders were invited due to their ability to disseminate information and shape normative beliefs and behavior through their leadership positions and social influence. In social marketing theory, these stakeholders are known as influence centers [27]. According to the diffusion of innovation theory, understanding where the attitudes of early adopters of a new idea or behaviors fall along the community readiness scale allow for a better understanding of strategies that are needed to move them into action and thus influence the wider community [19,28]. All invited participants were professional people aged 30–65 years who were involved with the prevention of family violence through either the CRE Reference Group, the CRE Community Champions or other anti-violence work such as White Ribbon work.

An analysis of responses from the initial focus groups generated the beginnings of a hypothesis that community attitudes were positive and pro-change, but knowledge of the causal pathways of FV necessary for implementing this change were lacking. Following this initial data analysis, other community members who were highly engaged with FV prevention or response were invited to attend a focus group. Thematic analysis was undertaken separately for each focus group and then across all focus groups. First, each focus group was manually transcribed and coded by the primary researcher. Emerging themes were analyzed, and member checking was undertaken to ensure the validity of results. Discussion of the emerging themes occurred among members of the research team, all of whom had different history and insights into the development of the CRE Plan. After the completion of data collection, all focus group transcripts were re-analyzed to triangulate themes that spanned the data and to identify outliers [29].

Ethics approval was obtained from the University of Western Australia prior to study commencement (RA/4/20/4860). A deliberative process of gaining verbal consent from all individuals to tape recording was obtained prior to the focus group/interview commencement. No incentive to participation was given beyond light refreshments. 

## 3. Results

Three focus groups were held. Only two people showed up for a fourth focus group, so a structured discussion was undertaken with them, and another two individuals interviews were undertaken with potential participants unable to make the focus groups. Data from these four additional informants were not included in the formal analysis, but the themes identified were concordant with those identified through the focus group analysis.

The focus groups consisted of one group of six women, one of five males, and one mixed gender group (four women and two men). A total of 35 people were invited to participate, with an attendance rate of 48.6%—or 17 people in attendance across the three focus groups (six, five and six participants, respectively). The first two focus groups consisted of members of the CRE Reference Group, an organizational stakeholder advisory group for the CRE Strategic Plan. Participants included professionals and community leaders aged between 30 and 60 years. As noted above, the sampling approach using community members and organizational leaders meant that they were not representative of the general population. While all participants were residents of Geraldton, recruitment was not from the general population, and youth were not included. Two of the 17 participants identified as Aboriginal (one man and one woman), similar to the percentage of Aboriginal people living in Geraldton. Two of the 17 participants were born overseas, around half of the percentage of people in Geraldton who were born overseas. A lack of time was the single largest factor in low numbers, and Focus Groups 2 and 3 had to be rescheduled twice to improve attendance. The proposed fourth focus group was planned as a mixed gender group, although only two people turned up on the day.

Though not the focus of the discussions, some personal experiences of FV did arise in the focus groups without prompting [30]. All participants were given information about helplines should the focus groups cause personal distress. 

Four key themes (Table 1) were identified through analysis and data triangulation [29] and are elaborated below.

### 3.1. A Silent Subject

Almost all focus group members agreed that there is currently little prevention of FV in Geraldton, with the majority of resources going towards response-focused activities such as the women’s refuge and policing. The idea that FV is a “silent subject,” as one participant put it, was evident from discomfort around discussion of the issue. Another participant commented “Family violence isn’t a ‘sexy’ subject,…people just do not want to talk about it” (Person 5, Focus Group (FG) 1, male). This was exemplified by one participant, who suggested that increasing comfort around talking about FV could be used to measure the success of the prevention program.

“Also, look at how comfortable people are with talking about it. I think now you’ll still find a high percentage of people who aren’t comfortable … but the more education gets out there and the less the shame factor works into it, people will be more comfortable and that would be a good indicator that you’re making change.”

[Person 4, FG 3, female]

Participants talked about shame as a key reason for discomfort in talking about FV, both historically and continuing into the present. One participant discussed what it was like to grow up in the 1970s around family violence, and that “it was very much pushed into you that you don’t talk about it, because no one will respect you anymore, no one will understand, you’ll be all by yourself ….” [Person 1, FG 3, female]. Unfortunately, this feeling of shame around the topic was not only historical, as it was echoed by participants when discussing the present day: “Shame keeps people at home, shame stops people talking, shame stops you taking your kids to playgroup” [Person 6, FG 3, female].

One participant noted that the silence acts to support FV: “It’s not all about the victim being the one responsible for change, but it’s about the perpetrators and the communities that support that by not saying anything about it” [Person 2, FG 3, female]. 

### 3.2. Preventing FV is Intergenerational Work

There was an understanding about the difficulty of overcoming FV and that there needed to be long term commitment to primary prevention approaches

Change has to start in small spaces, which will make generational changes. There’s no quick fixes, but these strategies do need to be put into place so that this generational change can start. But we do need to accept that it won’t change overnight and it will be very, very hard work and lots of people need to be working together.

[Person 5, FG 2, male.]

While there was recognition that things are “better than they were 20 years ago” [Person 5, FG 3, male], all participants acknowledged that preventing FV is intergenerational work that will take long term, sustained funding and support. Participants acknowledged that “we’ve made massive progress in this space” [Person 5, FG 1, female] when compared with one to two generations ago, while also noting how far we have yet to go. Change is happening because “today’s young people, … how they see the boys at school and how the boys at school see them is vastly different than when we were growing up or even when my daughter was growing up” [Person 6, FG 3, female], but change was seen to be slow and hard won.

Moving forward, the participants identified the need for broad-based education as essential for success, along with persistence, leadership, follow-up, and organizational support. Barriers to organizational change were identified as organizational hierarchy, the fear of a “tokenistic gesture,” and the need to get the whole workplace involved, as it was felt that “there is no point unless everyone in the workplace agrees” [Person 2, FG 2, male]. A key barrier to change at the community level was seen to be the normalization of FV; it was mentioned several times that this normalization is challenging to change and to bring awareness to: “if you’re born into it, it’s the norm” [Person 1, FG 2, male].

In the men’s specific focus group, the attitude that FV is yet another ‘issue’ was discussed, with responses ranging from hopeful to exhausted. Participants were aware of the work necessary, but some felt overwhelmed by the enormity of the task ahead in the face of so many competing ‘causes.’ This suggests that motivation and collaboration to take the strain off any single organization will be necessary for this intergenerational work to be successfully completed while avoiding the desensitization and fatigue that was discussed in the men’s focus group. 

### 3.3. Who Needs to be in the Room?

The need to involve victim/survivors and perpetrators in changing the conversation was also a key theme. The authenticity that comes from lived experience was seen as an important means of driving change.

….the people that have actually experienced this are not in the room. We can throw the theory at it, but to get the answers we need to involve those who have experienced it.

[Person 1, FG 2, male]

Several participants commented on the need to involve men in prevention work (“Actually, it’s a man’s problem”) [Person 2, FG 1 female], and one participant suggested that the level of involvement of men could be a measure of the success of the strategy.

I really think we need that level of engagement with our young men in the town, because right now the victims have really taken ownership of trying to prevent these things, but we also need the perpetrators on the other side.

[Person 1, FG 3, female]

There was also a discussion around the need to engage the community with education efforts across the lifespan, with a specific target on childhood. This focus on early education around family violence prevention and gender equality work was known to be already occurring in Victoria where the government is implementing respectful relationships across Victorian schools and early childhood services (Victoria State Government, 2019), but such implementation has not as yet been implemented in WA.

### 3.4. Understanding the Role of Gender Inequality

Change the Story makes a clear distinction between gendered drivers and reinforcing factors for FV. Despite their proximity to the CRE Plan, which was based upon the Change the Story framework, it was clear that many participants did not understand or acknowledge the distinction and saw the reinforcing factors as causes of FV.

Drugs and alcohol are the common denominator.

[Person 5, FG 2, male]

I’ve spent the last two years volunteering with the [local program] … and some of the stories that our girls have, they’re experiencing FV because of drug use, and incidents have been increasing due to reporting, but also severity since meth has hit the street. That has definitely had a devastating effect.”

[Person 1, FG 3, female]

Lots of people want to be good, but there’s key factors like can’t find a job, stuck in a rut, stuck on Centrelink, stuck in influences around the neighborhood, etc.

[Person 3, FG 2, male]

This was a recurring theme in every focus group and discussion. When asked what causes or drives FV, almost all participants, barring those directly involved in the creation of the CRE Prevention Strategy, began to discuss what the Change the Story framework describes as reinforcing factors, that is, factors that are not causal but that often co-occur with FV or that, when present, increase the severity or frequency of FV [12,13]. The concept of gender inequality as a driver and these other factors as reinforcing rather than causal is a key aspect of the evidence-based implementation of FV primary prevention initiatives. However, gender inequality being causal was understood poorly or not at all, even in this highly engaged subpopulation [17,31]. In the focus groups, alcohol and drugs were the most commonly named “cause” of FV followed by unemployment, general poverty, lack of housing, stress, mental illness, structural oppression, and geographic isolation. This focus on more proximal and visible contributors to a problem may also reflect that gender inequality is pervasive, and, although it is a strong determinant, it is less evident than other causes due to its omnipresence. 

Of the four primary drivers of FV, focus group participants recognized the condoning of violence against women and male peer relations that emphasize aggression and disrespect towards women as problematic.

I also think there’s an emerging conversation around how young men and communities treat women or people experiencing family violence.

[Person 2, FG 3, female]

I think the other thing organizations can do is model behaviors at work. I know generally organizations don’t hit women, but in terms of respect in the workplace, like what sort of language you’ll tolerate and what sort of jokes you’ll tolerate. Those sorts of things…

[Person 5, FG 3, male]

Participants showed an emerging understanding of the impact of men’s control of decision-making and limits to women’s independence in public and private life, as well as rigid gender roles and stereotyped constructions of masculinity and femininity.

I think it’s also part of being a regional area, those old traditional sort of values are, I don’t want to say stronger, but more prevalent. I know in my upbringing, there’s no FV whatsoever, but I look to my father instinctively as the leader of the house. He’s the one we follow, regardless of his example, that’s who we follow. And then being in a regional area I feel we have that sort of stronger tie to these values.

[Person 4, FG 3, female]

The recognition of traditional gender roles in the context of FV gives hope that the idea is taking root. However, to gain traction in the community and abide by best practice, key stakeholders need to become comfortable explicitly discussing the causal association of gender inequality with FV [17,31,32]. 

Additionally hopeful was the recognition of power and control as a perceived factor in FV.

It’s a control sort of thing. I wonder what it is that makes people crave that kind of control over their partner, maybe it comes from a chaotic sort of life, where the one thing I can control is my power over you.

[Person 2, FG 2, male]

One person noted the early and very distressing impact of violence on Aboriginal women.

The 13 year old Aboriginal girl is the lowest person in any community, you don’t get any worse than being a 13 year old Aboriginal girl, it’s horrendous for them.

[Person 6, FG 3, female]

Another participant noted the impact of colonization on the unequal burden of FV in Aboriginal communities.

It’s the history of this country, of Australia as a whole. It started violently and it still seeps through. There’s trauma that’s unaddressed, both with Aboriginal people and non-Aboriginal people, and that keeps replaying and coming up each time.

[Person 5, FG 1, female]

This emphasizes the importance discussed above of understanding the intersection of the ongoing impact of colonization on Aboriginal people with the gendered drivers described in Change the Story.

## 4. Discussion

The community readiness model aims to assess how ready a community is for change to occur; this group of highly engaged participants provides insight into overall community readiness. This is because the readiness for change in this group of early adopters will necessarily be higher than the overall community due to their previous engagement; thus, their views can inform prevention efforts at all levels of the community. Focus group participants who were further from the locus of planning around prevention efforts tended to align more closely with the lower ends of community readiness. Participants acknowledged FV as a serious problem and as common in the community, but in terms of conscious change, only a few had a comprehensive knowledge of prevention theory and efforts to address it [19,21]. In these early stages, leaders and community members believe that FV is an issue for the community, but there is no immediate intent to act, and the decisional balance is tipped towards the negatives. This tilted decisional balance is seen in the plethora of perceived barriers to implementation that were mentioned in the focus groups, such as organizations being too small/too big, having too much hierarchy, an inability to make policy changes, not enough time, external management, difficulties in understanding the prevention efforts, worries about backlash, macho workplace values, and a lack of workplace buy-in. Of note, Kelly and colleagues have stated that identifying barriers is an important stage in that it indicates thinking about the need to raise awareness and develop concrete ideas to address the problem [19].

Certain key organizational stakeholders were more progressed, at or beyond the planning phase, recognizing a strong impetus for change but impeded by limited resources and without detailed and focused plans or efforts to address FV. Participants understood the difficulty of the task and many of the specific challenges. They also had important suggestions around strategy implementation and evaluation, including increasing the participation of men, reducing silence and shame about the subject of FV, and bringing the experiences of victim/survivors and perpetrators into the conversation. 

Overall, community attitudes in this subpopulation are positive and pro-change, but the knowledge of causal pathways of FV is lacking. While all participants agreed that FV is a problem in the community, many do not see gender inequality as the main driver of FV. Participants often had trouble understanding what a community that prevents FV would look like due to a lack of knowledge about what primary prevention entails. Participants were clear about ‘changing the conversation’ as a metaphor for preventing FV, and they had suggestions about who should be involved, what ‘the conversation’ should include and how prevention efforts could be evaluated. However, the conceptual link between increasing gender equality and preventing FV is not well understood, possibly due to the CRE Plan’s lack of focus on this driver of FV. Continued education at all levels of the community is necessary to help service providers and leaders conceptualize what prevention would look like, to engage community members in a conversation that takes FV beyond the simplistic victim/perpetrator dichotomy, and to change the story for regional women. 

The most common misunderstanding around the primary cause of FV revolves around alcohol and drugs. The main argument against alcohol and drug use as a primary cause of FV is that it cannot explain the highly gendered patterns of violence perpetration and victimization. Identifying alcohol and drug use as a primary cause of FV can also work to excuse violence, thus reinforcing the culture which condones it. 

The rates of harm from alcohol are considerably higher in rural than metropolitan areas, with rural residents more likely to engage in alcohol consumption that exceeds thresholds for short-term harm and long-term harm [33]. With both high rates of FV and high rates of alcohol misuse, it is not surprising there is considerable overlap. Unfortunately, the misunderstanding that alcohol and drugs “cause” FV has proven to be difficult to change. One study found that the percentage of people believing that alcohol consumption is the single largest predictor of FV actually increased following training that did not advocate this view [34]. This may be due to the phenomenon described by cognitive science, that when facts are presented in a manner than is incongruent with a person’s values, the facts will be rejected, not the values framework within which the person is functioning [35]. This model recognizes that communication is not a purely rational endeavor; rather it theorizes that the frameworks of values and norms that each individual functions within have a large impact on their understanding and acceptance of facts. For someone to accept new facts, they must be presented in a way that is not incongruent with their values systems [36,37]. This points to a key challenge of a primary prevention strategy that aims to change a deeply ingrained value system. Only by understanding resistance to change can we determine how best to engage the population and refocus the conversation on primary prevention. 

Another barrier to seeing gender inequality as a primary cause of FV may be that in the response-focused world of FV services providers are engaging with reinforcing (exacerbating) factors such as alcohol/drug use and mental health issues on a daily basis, while prevention remains abstract and not achievable in the foreseeable future. In the focus groups, gender inequality was rarely directly broached. The topics of men’s control of decision-making and limits to women’s independence in public and private life, rigid gender roles, and stereotyped constructions of masculinity and femininity were barely touched on. This shows there is considerable work to do in Geraldton and much to learn from approaches being undertaken elsewhere. There has been increasing attention on the role of men leading work in violence against women, promoting gender equality and expanded roles for fathers [16,38,39]. Having men as role models in leading the fight against paternalism and challenging male privilege creates a particularly powerful alliance in achieving change but also has considerable challenges [40]. However, it is worth noting that a Men Against Violence initiative was implemented in Geraldton within a few months of the focus groups being held and received considerable support from key stakeholders.

To change the current status and achieve functional education for the whole population, engagement with the prevention of FV needs to increase at all levels of the community, from the highly supportive and well-informed service provider to community members who have not yet begun to think about this issue. The CRE Strategy document outlines a clear plan of actions needed for primary prevention, including addressing gender inequality, and many individuals who participated in this study showed willingness and readiness to continue the challenging work of examining and re-evaluating their core values. 

Participants understood that achieving change was likely to be intergenerational in terms of being slow and needing to be sustained. The participants also understood that children who are exposed to violence are more likely to see this as a norm, but there was less explicit acknowledgement of the transmission of sexist attitudes from father to son, such as when sons hear their fathers verbally abusing their mothers. Thus, the work is also intergenerational because it needs to occur across generations since the problem is deeply rooted in modelled behavior and sexism transmitted in the family. 

Given that this group of community leaders are at varying stages in their individual readiness, which was reflected in their perception of community readiness, we recommend that gender inequality be explicitly discussed as a causal factor in FV education programs. This must include men’s control of decision-making and limits to women’s independence in public and private life, rigid gender roles, and stereotyped constructions of masculinity and femininity. Without these elements, any education risks reinforcing the very ideas it wishes to correct [34]. However, these discussions must be carefully framed to focus on the potential positive effects of gender equality for men as well as for women. This approach, that will free people from conformity to rigid gender roles and gender stereotypes, may help to avoid increasing the well-documented backlash that can arise from poorly framed discussion [36,37]. There is evidence that an organizational intervention that explicitly focusses on changing gender-bias habits can be effective in increasing the consciousness around gender bias and taking actions to promote gender equity [41], and such an approach could potentially complement the activities of the CRE Plan.

We recommend expanding efforts at community education and we recommend that these must use a variety of different means to engage with people across diverse population groups. This community education must occur at multiple levels and be interactive and ongoing to combat knowledge loss over time. It must be staged, as in the CRE Plan, to reduce backlash (as much as practicable). Discussing gender inequality is sometimes avoided early on in prevention strategies to avoid putting people ‘off side’ and thus increasing backlash; however, this risks slowing the rate of change and can reinforce misconceptions about causal pathways. This study shows the risk in avoiding discussions of causal pathways and recommends targeting education efforts at both the individual’s and the community’s readiness for change through multi-level efforts across the community. 

There are several limitations of this study, particularly related to the diversity of participants and small number of focus groups and individuals involved. Focus groups can be useful for enabling a rich discussion as individuals are catalyzed by the comments of others or expand or disagree with comments made by others. Focus groups can be constrained by social desirability bias or when dominant voices limit the willingness of others to participate. While some may criticize a model that emphasizes gender inequality as the basis for family violence, the Australian Government has accepted that is not possible to end violence against women and their children without addressing the systemic inequalities of power between men and women, as well as the narrow and stereotypical gender roles that are part of these inequalities [13]. Despite the narrow criteria for selecting the participants and that they were not representative of the wider population of Geraldton, it is likely that the themes that emerged would be replicated in other populations who are early in the process of implementing primary prevention initiatives against FV. 

## 5. Conclusions

There was considerable interest in the prevention of family violence in this regional city setting that experiences high rates of FV. It was surprising that, given strenuous efforts to engage the community and the participants in this study around FV prevention, a greater understanding of the drivers of FV and readiness to change were not apparent among this highly connected group of community leaders. What was missing was a good understanding of gender inequality as the key underpinning driver of FV. This suggests that more intensive education about the drivers of FV is needed. Public education must focus on dispelling misunderstandings of reinforcing factors being the cause of FV.

## Figures and Tables

**Table 1:** Key themes identified.

Theme	Elaboration
Silent Subject	Many participants spoke of FV as being taboo or a ‘silent subject’ and applauded efforts to ‘change the conversation.’
Preventing FV is difficult, intergenerational work	There was widespread acknowledgement of the extensive time and support necessary to prevent FV. At the organizational level, persistence, leadership, follow-up, achieving broad based support, and education about primary prevention were identified as necessary for success. At the community level, challenging the normalization of FV was seen as a high priority.
Who needs to be in the conversation	Engaging men and including victim/survivors and perpetrators were seen as important elements of the strategy to change the conversation. Education across the lifespan, especially including children, was also seen as essential.
Understanding the role of gender inequality in FV	It was acknowledged that the conversation about FV needs to change, but there was less awareness of causal pathways. Many participants named alcohol and drug use as the most common drivers of FV, though this is at odds with the theoretical underpinnings of the National Plan and the CRE Plan, which see alcohol and drugs as reinforcing factors.

## Appendix A

Focus Group Questions

1. What is your current knowledge of the CRE Strategic Action Plan?

a. What do you see as the goal of the CRE Plan?

2. How do you think Geraldton is doing at preventing family violence?

a. In your opinion, what drives family violence?

3. What do you think would be measures by which we would know the drivers of family violence are changing?

a. How might workplaces and researchers collaborate to gather this data?

4. What are any barriers/challenges that you see to implementing the CRE Action Plan in your workplace?

a. Beyond what is dictated by the CRE Plan, how might your workplace act to decrease the drivers of family violence and incidence of family violence in Geraldton?
